# A Review of Recent Hardware and Software Advances in GPU-Accelerated Edge-Computing Single-Board Computers (SBCs) for Computer Vision

**DOI:** 10.3390/s24154830

**Published:** 2024-07-25

**Authors:** Umair Iqbal, Tim Davies, Pascal Perez

**Affiliations:** 1 SMART Infrastructure Facility, University of Wollongong, Wollongong, NSW 2522, Australia; tdavies@uow.edu.au; 2 Australian Urban Research Infrastructure Network (AURIN), University of Melbourne, Melbourne, VIC 3052, Australia; pascal.perez@unimelb.edu.au

**Keywords:** computer vision (CV), edge computing, Internet of things (IoT), graphical processing unit (GPU), single-board computers (SBCs), smart cities

## Abstract

Computer Vision (CV) has become increasingly important for Single-Board Computers (SBCs) due to their widespread deployment in addressing real-world problems. Specifically, in the context of smart cities, there is an emerging trend of developing end-to-end video analytics solutions designed to address urban challenges such as traffic management, disaster response, and waste management. However, deploying CV solutions on SBCs presents several pressing challenges (e.g., limited computation power, inefficient energy management, and real-time processing needs) hindering their use at scale. Graphical Processing Units (GPUs) and software-level developments have emerged recently in addressing these challenges to enable the elevated performance of SBCs; however, it is still an active area of research. There is a gap in the literature for a comprehensive review of such recent and rapidly evolving advancements on both software and hardware fronts. The presented review provides a detailed overview of the existing GPU-accelerated edge-computing SBCs and software advancements including algorithm optimization techniques, packages, development frameworks, and hardware deployment specific packages. This review provides a subjective comparative analysis based on critical factors to help applied Artificial Intelligence (AI) researchers in demonstrating the existing state of the art and selecting the best suited combinations for their specific use-case. At the end, the paper also discusses potential limitations of the existing SBCs and highlights the future research directions in this domain.

## 1. Introduction

Computer Vision (CV), the interdisciplinary field that enables machines to extract information from visual data, has witnessed significant advancements in recent years [1,2,3]. The availability of high-resolution cameras, coupled with the exponential growth in computational power, has opened up new opportunities for developing CV solutions across a wide range of domains [4,5,6]. From object detection and recognition to image segmentation and scene understanding, CV plays an important role in enabling machines to perceive and interpret visual information. Considering smart cities as a use case, CV has been used to address the urban challenges such as traffic management [7], waste management [8], transport safety, [9] and flood management [10]. However, the increasing complexity and scale of modern CV applications pose challenges in terms of computational resources and latency [11,12]. Traditional centralized computing approaches, where data is processed in the cloud or remote servers, struggle to meet the stringent real-time requirements of many CV applications. The reliance on cloud-based processing introduces inherent limitations such as network latency, bandwidth constraints, and potential privacy and security concerns [13,14].

To overcome these limitations, edge computing has emerged as a promising paradigm for deploying CV applications. Edge computing brings the processing capabilities closer to the data source, enabling real-time analysis and decision making at the edge of the network [15,16]. By leveraging local processing power, edge computing reduces the reliance on the cloud, mitigates latency issues, and improves the overall system performance. In addition, it improves on data privacy, since no real data is being stored or transmitted, rather only the post-algorithmic analysis is being communicated, making it a suitable option for problems involving ethics [16].

Graphical Processing Units (GPUs) have played a significant role in advancing the field of CV, particularly in deep learning-based approaches [17,18]. GPUs are highly parallel processors designed for rendering graphics, but their architecture and computational power have made them indispensable for accelerating a wide range of compute-intensive tasks, including CV algorithms. The parallel nature of GPUs allows for the efficient execution of matrix operations and Convolutional Neural Networks (CNNs), which are fundamental to many CV tasks [18]. In recent years, GPUs have seen remarkable advancements in both hardware and software, enabling their seamless integration into edge-computing environments and Single-Board Computers (SBCs) [19,20]. Hardware improvements include enhanced GPU architectures with increased parallelism, higher memory capacity, and improved power efficiency, making them compatible with the resource-constrained SBCs commonly used in various edge-computing applications. These advancements have enabled GPUs to handle more complex and sophisticated CV algorithms in real time to address real-world problems, making them a preferred choice for edge devices with limited resources. On the software front, various frameworks and libraries have emerged to harness the power of GPUs for accelerating CV tasks. NVIDIA’s Compute Unified Device Architecture (CUDA) framework has become a widely adopted solution, providing a programming model and runtime system for GPU-accelerated computing [21].

The integration of GPUs with edge computing opens up new possibilities for real-time CV applications. The ability to process visual data locally on edge devices, such as cameras, drones, or autonomous vehicles, enables timely decision making and reduced dependence on cloud infrastructure. Furthermore, it enables privacy-sensitive applications where sensitive data can be processed locally, minimizing the need for data transmission to external servers. However, there are challenges associated with GPU-accelerated edge computing for CV. One key challenge is the effective utilization of limited computational resources on edge devices, considering the trade-off between model complexity and hardware constraints. Another challenge lies in balancing the computational workload between the edge device and the cloud, taking into account factors such as network bandwidth, latency, and cost. Additionally, the integration of other sensor technologies, such as Light Detection and Ranging (LiDAR) or depth cameras, with GPU-accelerated edge computing presents further opportunities and challenges for real-time perception tasks. There exists a gap in the literature to comprehensively discuss the existing hardware and software platforms for GPU-accelerated CV development on SBCs.

This review paper aims to provide a detailed overview of the recent hardware and software advances in GPU-accelerated edge-computing SBCs for CV. By examining the latest developments in GPU-based hardware, software frameworks, software packages, existing limitations, and future opportunities, this review seeks to help readers in better understanding the technological advancements in this domain over the years and be informed about the challenges. Furthermore, it provides a subjective comparative analysis of the technologies and cross-platform mapping to help the reader decide on the selection of technologies corresponding to a specific category of SBCs for a custom use case.

## 2. Review Taxonomy

This review is one of its kind and different from conventional reviews where the focus is more towards purely research-based advancements. This review is aimed more towards highlighting the advancements in the applied Artificial Intelligence (AI) domain, specifically for GPU-accelerated hardware and software advancements in deploying CV based/video analytics solutions on SBCs for onboard real-time processing. This makes the scope of this review very specific, only limited to the edge-computing deployment of CV solutions. Further, this review is aimed to enlighten readers about the clear understanding about how the different technologies in this context have evolved and what is the current state of the art. It is also important for an applied AI scientist to know the existing challenges and limitations of the relevant technologies. At the end, future research directions are also highlighted. This review is structured as follows: first, a detailed description of how GPU-accelerated hardware evolved over the years and categorizing the hardware based on the leading manufacturers. A comparative table is also provided. In addition, the existing SBCs are mapped against the fundamental CV tasks based on the technical specifications and requirements of the CV task. The next section discusses software developments including what packages have been used for development, what frameworks are being built on top of fundamental packages, what tools/packages are specifically designed for edge-computing or onboard deployment, and what different model optimization techniques are used for deployment onboard. At the end, the challenges and limitations are highlighted along with future research directions.

## 3. Advances in GPU-Accelerated Hardware

Given the advancements in electronics technology and on the affordable commercial rates, the demand of SBCs increased over the years. There are three main manufacturers of GPU-accelerated SBCs: NVIDIA (Santa Clara, CA, USA), ASUS (Beitou District, Taipei, Taiwan), and Libre (Hua District, Shenzhen, China), each having a range of edge computers based on the utilities and price range. NVIDIA is leading with the highest number of GPU-accelerated products to facilitate entry-level and high-performance processing applications, suitable for all. This section presents the details of the most common and emerging SBCs available in the market and also provides a comparative analysis.

### 3.1. ASUS Tinker Boards

ASUS has developed a range of GPU-accelerated SBCs in the recent past to meet the computing needs of various professionals. The series includes the Tinker Edge T (released in 2019); the Tinker Board 2 (released in 2020); the Tinker Board S (released in 2021); and the latest Tinker Board 3 N (released in 2023). The Tinker Board S features a quad-core Advanced RISC Machine (ARM)-based Central Processing Unit (CPU) (Rockchip RK3288-CG W), paired with an ARM Mali-T764 GPU. This setup is suitable for media playback, CV tasks, image processing, and light gaming. Conversely, the Tinker Edge T is equipped with an NXP i.MX 8 M quad-core ARM-based CPU and ARM Mali-T860 GPU, enhanced by the integration of a Google Edge Tensor Processing Unit (TPU), making it adept at Machine Learning (ML) inference tasks. The Tinker Board 2 is equipped with a hexa-core Rockchip RK3399 CPU utilizing ARM’s 64-bit ARMv8 architecture and an ARM Mali-T860 GPU. This combination offers improved computational performance, suitable for more demanding tasks. Lastly, the Tinker Board 3 N features a quad-core Rockchip RK3568 CPU and an ARM Mali G52 GPU, providing enhanced computational and graphics rendering capabilities.

In terms of memory and storage, the Tinker Board S includes 2 GB of Random Access Memory (RAM) and a built-in 16 GB embedded Multi-Media Card (eMMC) module, with an option to expand via a Micro Secure Digital (microSD) card. The Tinker Edge T, although slightly lower in RAM with 1 GB of Low-Power Double Data Rate (LPDDR)v4, includes 8 GB of eMMC storage and also supports microSD expansion. The Tinker Board 2 matches the Tinker Board S with 2 GB of LPDDR4 RAM and adds a 16 GB eMMC module. The Tinker Board 3 N stands out with 4 GB of LPDDR4 RAM and 64 GB of built-in eMMC storage, ensuring enough memory and storage capacity for more complex applications. In terms of Input/Output (I/O) connectivity, the Tinker Board S features a 40-pin General-Purpose Input/Output (GPIO) interface, Display Serial Interface (DSI), Camera Serial Interface (CSI), High-Definition Multimedia Interface (HDMI) output, and Universal Serial Bus (USB) Type-A ports. The Tinker Edge T offers in addition USB Type-C, Gigabit Local Area Network (LAN), Wi-Fi, and Bluetooth. The Tinker Board 2 includes HDMI, DSI, USB Type-C, CSI, 40-pin standard connector, USB Type-A, Wi-Fi, and Bluetooth support, enhancing its versatility. The Tinker Board 3 N provides HDMI, USB Type-C, USB Type-A, Wi-Fi, and Bluetooth, ensuring robust connectivity for modern applications.

In terms of form factor, the Tinker Board S and Tinker Board 2 both measure approximately 3.37″ × 2.125″ and weigh 55 g, making them compact and portable. The Tinker Edge T is slightly larger at 3.35″ × 2.20″ but maintains the same weight. The Tinker Board 3 N, although the heaviest at 60 g, has a slightly larger footprint at 4″ × 4″. Power consumption varies across the boards: the Tinker Board S consumes around 5 W, the Tinker Edge T ranges from 5 W to 10 W, the Tinker Board 2 is between 5 W to 10 W, and the Tinker Board 3 N has a higher consumption of 10 W to 15 W. The pricing of these boards reflects their capabilities and target audiences. The Tinker Board S is available at USD 199 [22], positioning it as an affordable option for developers needing a versatile SBC. The Tinker Edge T, priced at USD 240 [23], targets developers requiring robust ML capabilities despite its higher cost. The Tinker Board 2 offers a good balance of performance and affordability at USD 120 [24], suitable for IoT projects. The Tinker Board 3 N, priced at USD 160 [25], provides a powerful solution for low-end computing tasks with ample storage and connectivity options.

In summary, the Tinker Board S, with its decent GPU, is attractive for general media tasks and light graphical processing but struggles with heavy multitasking due to its limited RAM and passive cooling. In the literature, this board is reported to be used for COCO detection [26], face detection [27], and face with mask classification [28] tasks. The Tinker Edge T excels in ML tasks with its Google Edge TPU but is less suited for heavy multitasking or real-time applications due to its low RAM and basic GPU. The literature reports the use of Tinker Edge T for pest classification [29] using a ResNet8 model. The Tinker Board 2 offers enhanced performance with its hexa-core CPU but also faces challenges with demanding applications due to its limited RAM and passive cooling. The Tinker Board 3 N stands out with its powerful CPU and substantial storage, making it a versatile choice for various applications, though its higher power consumption and passive cooling may limit its suitability for low-power or sustained high-load tasks.

### 3.2. NVIDIA Jetson Boards

NVIDIA is one of the leading manufacturers of the GPU-accelerated SBCs and introduced cutting-edge technologies for meeting the computational needs of the latest deep learning models. Over the years, NVIDIA has introduced a range of SBCs including Jetson Nano (released in 2019), Jetson TX2 (released in 2017), Jetson Xavier NX (released in 2020), Jetson AGX Orin (released in 2023), and Jetson Orin Nano (released in 2023). The GPUs on these boards are designed to handle various levels of AI workloads. The Jetson AGX Orin features an Ampere architecture GPU with 2048 CUDA cores and 64 Tensor cores, offering top-tier performance. The Jetson Xavier NX uses a Volta architecture GPU with 348 CUDA cores and 48 Tensor cores. The Jetson TX2 is equipped with a Pascal architecture GPU with 256 CUDA cores, and the Jetson Nano uses a Maxwell architecture GPU with 128 cores. The Jetson Orin Nano, although an entry-level board, features an Ampere architecture GPU with 1024 CUDA cores and 32 Tensor cores, providing significant performance for being an entry-level board. In terms of CPUs, the Jetson AGX Orin is powered by a 12-core ARM Cortex-A78AE v8.2 64-bit CPU, while the Jetson Xavier NX features a hexa-core NVIDIA Carmel ARM v8.2 64-bit CPU. The Jetson TX2 has a dual-core Denver 2 64-bit CPU paired with a quad-core ARM Cortex-A57 MPCore. The Jetson Nano and Jetson Orin Nano both have quad-core ARM Cortex-A78AE v8.2 64-bit CPUs, making them efficient for their targeted applications.

In terms of compute power, the Jetson AGX Orin stands out as the most powerful, delivering up to 275 TOPS of AI performance. Following closely is the Jetson Xavier NX, capable of 21 TOPS, making it suitable for high-performance edge-computing solutions. The Jetson Nano and Jetson Orin Nano are more modest, designed for entry-level AI applications, while the Jetson TX2, with its AI computing capability, serves as a mid-range option. Memory capacity is another differentiator among these boards. The Jetson AGX Orin leads with 64 GB of LPDDR5 RAM, followed by the Jetson Xavier NX and Jetson TX2, each with 8 GB of RAM. The Jetson Nano and Jetson Orin Nano both come with 4 GB of LPDDR4 RAM. Storage options include built-in eMMC modules, ranging from 16 GB in the Jetson Nano to 64 GB in the Jetson AGX Orin. Additional storage can be expanded via microSD slots, especially useful for entry-level boards like the Jetson Nano and Jetson Orin Nano.

Connectivity options across the Jetson boards are robust. Common features include HDMI outputs, USB Type-A and Type-C ports, and various interfaces such as CSI, GPIO, and PCIe. The Jetson AGX Orin, with its extensive I/O connectivity including PCIe Gen4, Gigabit Ethernet, DisplayPort, and a 40-pin header, provides the most comprehensive connectivity options. The Jetson Xavier NX and Jetson TX2 also offer versatile connectivity, supporting Ethernet, Wi-Fi, Bluetooth, and multiple camera interfaces. Form factor and power consumption are crucial considerations for different applications. The Jetson Nano and Jetson Xavier NX are compact and lightweight, making them suitable for portable projects. The Jetson AGX Orin, although powerful, is larger and heavier, with dimensions of 4.33″ × 4.33″. The Jetson Nano and Jetson Xavier NX, on the other hand, are the most compact in the line up with dimensions of 2.72″ × 1.77″ and 2.74″ × 1.77″, respectively.

Power consumption varies significantly across different boards based on the compute resource and GPU technology. The Jetson Nano ranges between 5 W and 10 W, the Jetson TX2 averages around 15 W, the Jetson Xavier NX ranges from 10 W to 30 W, and the Jetson AGX Orin operates between 15 W and 60 W. The Jetson Orin Nano, balancing performance and efficiency, consumes between 7 W and 15 W. In terms of pricing, the Jetson Nano, priced at USD 249 [30], is an affordable option for educational and entry-level AI tasks. The Jetson TX2, at USD 810 [31], targets mid-range applications requiring higher performance. The Jetson Xavier NX, priced at USD 530 [32], offers a balance of cost and performance for high-performance edge-computing solutions. The Jetson AGX Orin, at USD 3000 [33], represents a significant investment for top-tier AI development capabilities. The Jetson Orin Nano, at USD 800 [34], provides a moderate option for medium-level computing applications.

In summary, the Jetson Nano is ideal for entry-level tasks but struggles with intensive workloads due to its lower compute power and memory. Furthermore, the passive cooling can become inadequate during intensive workloads, affecting sustained performance. In the literature, the Jetson Nano is reported to be used for pedestrian detection [35,36,37], ImageNet classification [38], MNIST classification [39], ship detection [40], face mask detection [41], DeepFashion2 classification [42], COCO detection [43], landing platform identification [44], person/weapon detection [45], plastic bag detection [8], and ripe coffee bean bunch detection [46]. The Jetson TX2 offers high performance but is less suitable for budget-sensitive projects. It supports active cooling using a fan making it suitable for long-term real-time tasks, however, at the expense of additional power consumption. The literature reports the use of TX2 for PASCAL VOC detection [47], COCO detection [26], DeepFashion2 classification [42], drone detection [48], vehicle detection [49], pedestrian detection [50], person detection [51], plastic bag detection [8], weed segmentation [52], fruit pest detection [53], and concrete crack detection [54]. The Jetson Xavier NX provides a good balance of cost and performance, though it requires effective cooling. Its moderate power consumption limits its use in battery-operated applications. Some examples of literature using NX include banana ripeness detection [55], face mask detection [56], COCO detection [43,57], vehicle detection [58], pedestrian detection [43,58], fire detection [59], and depth estimation [60]. The Jetson AGX Orin is a powerhouse for intensive AI workloads but is expensive and power hungry. It comes with active cooling through the fan, making it suitable for resource exhaustive real-time tasks. A few of the use-case applications where ORIN AGX is used include tomato disease classification [61], COCO detection [62], ground vehicle detection [63], surgical instrument detection [64], and vehicle detection [65]. The Jetson Orin Nano offers a balanced performance at a mid-range price, though its power consumption and cooling needs may limit its use in energy-sensitive applications. Some example use cases reported in the literature where the Jetson ORIN Nano is deployed include tomato disease classification [61] and vehicle detection [65].

### 3.3. Libre Boards

Libre is another leading competitor in developing low-cost SBCs for entry-level AI applications. A few common SBCs from the Libre series include the Libre Tritium (released in 2018); Libre Le Potato (released in 2017); and the Libre Renegade (released in 2018). In terms of computing power and CPU, the Libre Tritium is geared toward entry-level applications, featuring a maximum 64-core CPU designed for basic computing tasks. Despite its modest processing power, it can handle simple AI computations with its integrated hex-core 3D GPU. The Le Potato, Libre Computer’s flagship product, boasts a 64-bit quad-core ARM Cortex-A53 CPU, providing more robust performance for entry-level tasks. The Renegade also features a quad-core ARM Cortex-A53 CPU, offering a balance of processing power suitable for a range of applications. In terms of GPU capabilities, the Libre Tritium is equipped with a hex-core 3D integrated GPU, enabling support for basic AI computations. Le Potato uses an ARM Mali-450 penta-core GPU, which, while modest, enhances its ability to handle graphical tasks. The Renegade also features an ARM Mali-450 GPU, offering similar graphical capabilities to Le Potato, but with added support for 4 K HDR, making it suitable for more demanding graphical applications.

Memory and storage options vary across the Libre boards. The Tritium comes with 2 GB of DDR3 RAM and supports external storage via a microSD card slot. Similarly, Le Potato includes 2 GB of DDR3 RAM and an optional microSD card slot for storage needs. The Renegade, however, stands out with its 4 GB of DDR4 RAM, providing enhanced memory capacity for more demanding applications. It also supports external storage via a microSD card slot, ensuring flexibility in storage expansion. In terms of I/O connectivity options, the Tritium offers versatile I/O integration with features such as a DVP parallel camera interface, fast Ethernet connectivity, 4 K HDMI, and USB Type-A ports. Le Potato includes standard I/O features like USB Type-A ports, fast Ethernet, TRRS CVBS/AV Jack, and HDMI, supporting both Linux- and Android-based systems. The Renegade provides extensive connectivity options, including USB Type-A (v2.0, v3.0), Gigabit Ethernet, TRRS CVBS/AV Jack, HDMI with 4 K HDR support, a 40-pin low-speed header, and various other headers for I2 S, PWM, I2C, UART, SPI, SPDIF, and ADC, making it highly versatile for various peripherals and networks.

The form factor of all three boards is compact, with dimensions measuring approximately 3.34″ × 2.20″, making them suitable for a variety of applications where space is a constraint. Power consumption is also a critical factor, with the Tritium and Le Potato consuming around 5 W on average, making them energy-efficient options for basic tasks. The Renegade, with its more extensive connectivity and higher performance, has a power consumption range of 5 W to 10 W. In terms of pricing, the Libre Tritium is priced at an affordable USD 35 [66], making it an attractive option for basic applications and educational purposes. Le Potato is similarly affordable at USD 30 [67], offering a low-cost solution compatible with Raspberry Pi accessories, making it an easy replacement or upgrade for Pi users. The Renegade, priced at USD 45 [68], provides a cost-effective solution for more demanding tasks, with extensive connectivity options.

In summary, the Tritium, with its low cost and basic I/O options, is ideal for educational purposes and simple tasks but lacks robust performance and AI/ML framework support. Le Potato, while affordable and compatible with Raspberry Pi accessories, is limited in performance and support for advanced frameworks, restricting it to light, non-intensive use. The Renegade, with its better connectivity and higher memory, offers a versatile solution but struggles under heavy loads due to its basic passive cooling and limited development ecosystem.

### 3.4. Other Boards

Apart from the above-mentioned boards from three main manufacturers, there are a few other GPU-accelerated SBCs in the market including VisionFive 2 (released in 2021), ROCK PI N10 (released in 2021), BeagleBone AI (released in 2019), HiKey970 (released in 2017), Coral Dev (released in 2019), and Coral Dev Mini (released in 2020). In terms of CPU capabilities, the VisionFive 2 features a StarFive JH7110 64-bit CPU, which, although not the most powerful, provides sufficient performance for general applications. The ROCK PI N10 features Rockchip RK3399Pro, combining a dual-core Cortex-A72 with a quad-core Cortex-A53 and ARM Mali T860MP4 GPU, delivering up to 3.0 TOPs of computing power. The BeagleBone AI features a Texas Instruments Sitara AM5729 SoC with dual ARM Cortex-A15 microprocessors, dual ARM Cortex-M4 co-processors, and additional DSPs and Embedded Vision Engines for enhanced performance. The HiKey970 boasts a HiSilicon Kirin 970 SoC with a dedicated Neural Processing Unit (NPU) and a combination of Cortex-A73 and Cortex-A53 CPUs for robust processing power. The Coral Dev Boards feature quad-core Cortex-A CPUs designed for AI applications, with the Dev Board utilizing a Cortex-A53 and Cortex-M4F, and the Mini using a Cortex-A35.

In terms of GPU, The VisionFive 2 includes an integrated BXE-4-32 GPU from Imagination Technologies, offering basic graphical performance. The ROCK PI N10’s ARM Mali T860MP4 GPU supports significant AI computing tasks with its high TOPs rating. The BeagleBone AI incorporates a dual-core PowerVR SGX544 3D GPU, suitable for advanced graphics and AI tasks. The HiKey970 features an ARM Mali-G72 MP12 GPU, facilitating enhanced graphics performance and AI capabilities. The Coral Dev Board and Dev Board Mini come with integrated GC7000 Lite and IMG PowerVR GE8300 GPUs, respectively, both optimized for AI inferencing tasks. In terms of memory, the VisionFive 2 comes with 8 GB LPDDR4 RAM and supports storage via a microSD card slot. The ROCK PI N10 offers 4 GB LPDDR3 RAM, with storage options including eMMC 5.1 and microSD. The BeagleBone AI includes 1 GB of RAM and 16 GB of eMMC onboard flash. The HiKey970 stands out with 6 GB of LPDDR4 X RAM and 64 GB of UFS 2.1 storage. The Coral Dev Board features 4 GB of LPDDR4 RAM and 8 GB of eMMC storage, while the Mini version offers 2 GB LPDDR3 RAM and 8 GB eMMC flash memory.

The VisionFive 2 provides a range of connectivity options including an M.2 connector, eMMC socket, Gigabit Ethernet, 40-pin GPIO, RJ45 Ethernet connector, and USB Type-A (v3.0, v2.0). The ROCK PI N10 includes USB Type-A (v3.0, v2.0), LAN, UART, SPI bus, I2C bus, PCM/I2S, SPDIF, PWM, ADC, and GPIO. The BeagleBone AI features USB Type-C, USB Type-A, Gigabit Ethernet, and 2.4/5 GHz Wi-Fi with Bluetooth. The HiKey970 offers Bluetooth, Wi-Fi, GPS, HDMI, MIPI/LCD port, MIPI port, a 40-pin low-speed expansion connector, and a 60-pin high-speed expansion connector. The Coral Dev Board includes Wi-Fi, Bluetooth, USB Type-C, USB Type-A (v3.0), Micro-B serial console, Gigabit Ethernet, HDMI, MIPI-DSI display connector, and MIPI-CSI2 camera connector. The Mini version offers similar connectivity with a focus on compactness. In terms of form factor and power consumption, The VisionFive 2 measures 3.93″ × 2.83″ and consumes around 10 W. The ROCK PI N10 has dimensions of 3.93″ × 3.93″ and a power consumption between 15–18 W. The BeagleBone AI measures 3.50″ × 2.12″ and consumes between 5–10 W. The HiKey970 is larger at 4.14″ × 3.93″ with a power consumption of 10–15 W. The Coral Dev Board measures 5.40″ × 3.90″ with a power consumption of around 5 W, while the Mini version is more compact at 2.52″ × 1.89″ with a power consumption of approximately 3 W. In regard to price, The VisionFive 2 is priced at USD 65 [69], offering a cost-effective solution for entry-level computing applications. The ROCK PI N10, at USD 199 [70], targets industrial AI applications with its high computing power. The BeagleBone AI, priced at USD 198 [71], is suitable for applications requiring enhanced computing capabilities. The HiKey970, available for USD 239, caters to developers seeking advanced AI capabilities. The Coral Dev Board, at USD 200, and the Mini version at USD 100, are designed for AI development and deployment, with the Mini focusing on affordability and compactness.

In summary, the VisionFive 2 is reasonably priced and offers decent performance for general applications but has limited mainstream support and can struggle with high-performance tasks due to basic passive cooling. In a technical blog post, VisionFive 2 is reported to be used for universal object detection [72]. The ROCK PI N10 provides robust performance for AI tasks but has high power consumption and cooling requirements, making it less suitable for energy-efficient applications. The BeagleBone AI offers high performance and extensive I/O, making it suitable for industrial applications, though its high power consumption and cooling needs limit its use in portable or low-power projects. In the literature, it is reported for the use of the MNIST classification task [73]. The HiKey970 excels in AI capabilities with its dedicated NPU but has limited development support and passive cooling, making it less versatile overall. Some example uses of HiKey970 reported in the literature include the PASCAL VOC detection [47] and MNIST classification [39]. The Coral Dev Board and Mini version are designed for fast ML inferencing with extensive I/O options, but their passive cooling might need enhancement for heavy tasks, and their development ecosystems are not as extensive as NVIDIA’s, limiting their versatility for certain applications. However, the optimized versions of models on the Coral Dev board reported enhanced performance. The literature reports the use of the Coral Dev Board for ImageNet classification [38], eye optical disc segmentation [74], tomato disease classification [61], people detection [45,75], and weapon detection [45] tasks.

### 3.5. Comparative Analysis

The evolution of GPU technology has seen remarkable advancements through various architectural generations, leading to increased performance, improved efficiency, and greater specialization (see Table 1). Starting with early-generation architectures like Mali T764 and Mali T860, there was significant progress with mid-range architectures such as Mali G72 and Adreno 630, which enhanced graphics capabilities for a variety of devices. At the same time, high-end architectures like Maxwell GPU, Volta GPU, Pascal GPU, and Ampere GPU marked a significant shift towards exceptional computational power, featuring specialized components like Tensor cores and ray tracing capabilities.

In the realm of processing units, early examples like the ARM Cortex-A7 and ARM Cortex-A53 were designed for power efficiency and affordability, suitable for entry-level devices. As technology advanced, mid-range architectures such as NXP i.MX 8M and Rockchip RK3288-CG W emerged, offering improved processing power and architectural enhancements for more demanding applications. More recently, modern architectures like Rockchip RK3399, Rockchip RK3568, and ARM Cortex-A78AE have brought a new level of computational performance, characterized by higher performance metrics, sophisticated design, and support for emerging fields like AI and ML.

**Figure 1:**
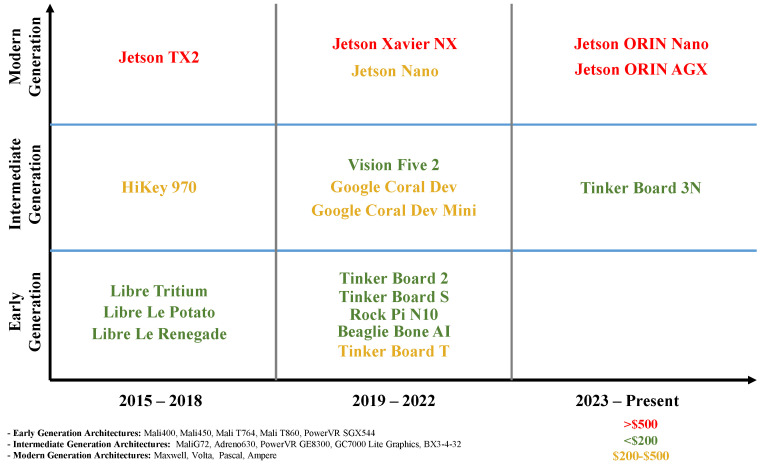
Cost and technology evolution analysis of existing GPU-accelerated Single-Board Computers (SBCs).

In summary, it can be reported that NVIDIA is the leading SBC manufacturer with dedicated/advanced GPU technology specifically to cater to the latest deep learning-based real-time solutions. They have introduced a range of product lines in terms of capabilities from Nano to Xavier NX to Orin AGX which covers low-end, intermediate-level, and high-level computational tasks for real-time deployment. However, the NVIDIA computers are very expensive and out of reach of students, other than their Nano model which is also becoming obsolete with the announcement of the Orin Nano (see Figure 1). On the other hand, for lower-end non-real-time CV tasks, ASUS Tinker Boards are an alternate option with relatively advanced architectures in comparison to others with reasonable pricing. All other boards are a bit outdated, and hardly available online for purchase (i.e., these were introduced in the market but could not compete) and only valid for lower-end applications. However, having said that, all the above remarks are subjective based on the limited information available. To confirm that a detailed benchmarking of existing hardware options needs to be performed for a standard task to better understand the possible utility of each item of hardware and where those can be used. At the end, Table 2 presents a summary of the application use cases reported in the literature where SBCs are deployed to address a CV task. This table compares the use cases in terms of CV tasks, the purpose of the use case, model, packages, and inference performance.

### 3.6. Mapping of Fundamental Computer Vision (CV) Tasks to SBCs

This section aims to align fundamental CV tasks with SBCs based on their computational capabilities. To achieve this, four distinct levels of CV tasks have been defined: entry-level, moderate-performance, high-performance, and very-high-performance tasks. These categorizations are founded upon considerations such as model complexity, visual task complexity, real-time processing demands, and related factors. Subsequently, corresponding to the hardware prerequisites for each category of task, suitable SBCs are recommended (see Table 3 for detailed comparison). This compilation serves as an informative resource to assist stakeholders in selecting appropriate hardware tailored to their specific application scenarios. It should be noted that the task classification presented herein is subjective and intended solely as a reference point for guiding decision making within the community.

#### 3.6.1. Entry-Level Computer Vision (CV) Task

An entry-level CV task typically involves employing a relatively simple deep learning model, such as MobileNet, in environments characterized by simple backgrounds with only objects of interest. Real-time processing is not a critical requirement, allowing for a few seconds per image during inference. Thus, hardware configurations with modest specifications suffice, such as a basic old-generation GPU, 2 GB of RAM, 16 GB of storage, and a 5–10 W power consumption allowance.

An illustrative example of such a task is the development of an image-classification system aimed at identifying common plant diseases from images of plant leaves. This application is intended to aid local gardeners and small-scale farmers in assessing the health status of their crops. The process involves capturing leaf images using a camera and conducting offline processing on an edge-computing device. The system does not necessitate rapid image processing; instead, periodic intervals between processing cycles are acceptable. For this purpose, SBCs such as Tinker Board S, Tinker Board T, Tinker Board 2, Libre Tritium, Libre Le Potato, Libre Renegade, ROCK PI N10, BeagleBone AI, and Coral Dev Mini are particularly suitable. These boards offer a cost-effective solution, with pricing ranging from USD 30 to USD 240, thereby aligning with budget constraints while meeting the computational demands of the envisioned CV task.

#### 3.6.2. Moderate-Performance Computer Vision (CV) Task

A moderate-performance CV task typically involves employing deep learning models of moderate complexity, such as ResNet50 pre-trained on the ImageNet dataset. The task requires inference times of less than one second per image and may involve processing images with some visual complexity (e.g., the presence of noise or irrelevant objects) in their backgrounds. To support such a task, suitable hardware would include a multi-core processor, an intermediate generation GPU with a minimum of 4 GB of RAM, 32 GB of storage, and a power consumption capability of around 10 W.

An illustrative example of a moderate-level task is the development of an image-classification system aimed at identifying animal species from images captured by animal trap cameras. This application serves the purpose of monitoring biodiversity and animal population dynamics. Typically, such tasks involve a larger number of classes, possibly up to 50, and require inference times comfortably under one second per image. For developing solutions for this type of task, SBCs such as Tinker Board 3 N, Jetson Nano, VisionFive 2, HiKey970, and Coral Dev Board are well suited. VisionFive 2 represents a cost-effective option (i.e., USD 65), while Jetson Nano offers enhanced functionality and performance capabilities.

#### 3.6.3. High-Performance Computer Vision (CV) Task

A high-performance CV task involves utilizing advanced object detection models like the YOLO series and Transformer-based DINO for real-time inference, typically requiring a minimum of 15 FPS and continuous operation in complex dynamically changing environments. To support such tasks, the hardware requirements include the latest generation CPU technology, a dedicated GPU architecture with Tensor cores, more than 16 GB of RAM, 64 GB of storage, and a power consumption capability of at least 15 W.

An exemplary application of such a task would be the development of a real-time traffic analytics system capable of detecting and counting various types of vehicles from a live camera feed. This system plays a crucial role in analysing traffic patterns and enhancing local traffic management strategies. It involves employing object detection models and tracking algorithms to accurately count distinct objects in real time. For implementing solutions for such high-performance applications, SBCs like Jetson TX2, Jetson Xavier NX, and Jetson Orin Nano are well suited. These devices are designed to meet the computational demands of real-time object detection and tracking tasks, however, at the higher end of the cost spectrum.

#### 3.6.4. Very-High-Performance Computer Vision (CV) Task

A very-high-performance CV task involves simultaneous inference of multiple models on multiple input streams in complex visual environments, achieving real-time processing speeds of over 15 FPS. To execute such tasks effectively, hardware requirements include state-of-the-art GPU architecture, a minimum of 32 GB RAM, 128 GB storage, and a power consumption capability exceeding 30 W.

An illustrative example of such a task is the development of a video analytics system designed to detect various types of vehicles, classify them based on colour, and count their unique occurrences across frames. This system must handle more than two input streams concurrently and deliver output at a rate exceeding 15 FPS. For implementing solutions for such demanding applications, Jetson AGX Orin is highly recommended. It is specifically designed to meet the computational demands of multiple model inference on complex video streams, ensuring high-performance real-time processing capabilities.

## 4. Software Advances

To facilitate the development of CV algorithms and solutions to be deployed on SBCs, over the years, a number of noticeable advancements have been made, specifically in terms of optimization approaches, development packages, development frameworks, and hardware deployment packages.

### 4.1. Computer Vision (CV) Algorithm Optimization Techniques

#### 4.1.1. Model Quantization

Model quantization involves reducing the precision of weights and activations in neural networks. Post-training quantization techniques, such as TensorFlow Lite’s post-training quantization [78], enable the conversion of models to more efficient 8-bit representations without retraining. Quantization-aware training techniques [79], such as training with mixed precision or using quantization-aware training frameworks like TensorFlow, PyTorch, and Open Neural Network Exchange (ONNX), allow for training models that can directly be quantized without significant loss in accuracy. Model quantization has emerged as a critical technique for optimizing deep learning models to meet the computational constraints of edge-computing devices. Over the years, various quantization approaches have evolved, enabling the deployment of efficient and lightweight CV models for edge-computing applications. Early model quantization techniques primarily focused on reducing model precision from 32-bit floating-point to 16-bit or 8-bit fixed-point representations, thereby reducing memory requirements and accelerating computations. However, these initial approaches often resulted in significant accuracy degradation, limiting their practical applicability. Advancements in model quantization techniques have addressed these challenges by incorporating sophisticated methods such as the following:

- Post-Training Quantization: This technique involves converting pre-trained models to low-precision representations, typically 8-bit fixed point, without retraining. Notable advancements include TensorFlow’s post-training quantization, which provides a straightforward approach for converting models to 8-bit precision, significantly reducing memory usage and accelerating inference without compromising accuracy [78].
- Quantization-Aware Training: This technique integrates quantization constraints during the model training process, enabling the creation of models that can be directly quantized without significant accuracy loss. Noteworthy advancements in quantization-aware training include the development of techniques in popular deep learning frameworks such as TensorFlow, PyTorch, and ONNX, allowing for the seamless integration of quantization during the training phase [78].

Recent developments in model quantization have also explored hybrid precision quantization, which combines different bit precisions for different model components to achieve a balance between model size and accuracy. Furthermore, research efforts have focused on optimizing quantization-aware training to better handle non-uniform quantization and mitigate the impact of quantization on model performance. Challenges still persist in achieving high levels of model compression while preserving the accuracy and robustness of CV models. As the demand for edge computing continues to grow, ongoing research is focused on developing novel techniques that enable more aggressive quantization without compromising the performance of CV models.

#### 4.1.2. Model Pruning

Model pruning involves identifying and removing redundant or less important parameters in neural networks [80]. Magnitude-based pruning methods, such as magnitude pruning and iterative pruning, focus on eliminating less significant weights [81]. Filter pruning methods, such as network slimming and ThiNet, target entire filters or channels for removal [82]. Structured pruning techniques, like weight sharing and structured sparsity, prune entire structures within the network [83]. Over the years, various pruning approaches have evolved, addressing the challenges of reducing model size while preserving accuracy and performance. Early techniques focused on eliminating redundant connections or weights within neural networks, aiming to reduce model size without a significant degradation in performance [80]. These methods included the following:

- Magnitude-Based Pruning: This approach involves removing small-magnitude weights or connections from the network, often based on a predefined threshold. The advancements in this technique have led to the development of iterative pruning methods, which iteratively prune the least significant weights and fine-tune the remaining network to maintain performance levels [81].
- Filter Pruning: Filter pruning techniques aim to remove entire filters or channels within convolutional layers that contribute minimally to the network’s overall output. Network slimming and ThiNet are examples of filter pruning techniques that have demonstrated significant reductions in model size while preserving model accuracy [82].

To address the limitations of early pruning methods and achieve more aggressive model compression, researchers have also developed advanced pruning strategies such as the following:

- Structured Pruning: Structured pruning techniques target specific structures within the network, including entire neurons or layers, for removal. By leveraging the structured patterns present in neural networks, these techniques enable more efficient and systematic model compression. Recent advancements in structured pruning have focused on preserving model performance through techniques such as gradual pruning and retraining, ensuring that the pruned networks retain their original functionality [80].
- Channel Pruning: Channel pruning techniques specifically target individual channels within convolutional layers based on their importance to the network’s output. These methods identify redundant or less significant channels and selectively prune them to reduce computational overhead while maintaining model accuracy. Recent developments in channel pruning have emphasized the integration of sparsity regularization and fine-grained pruning techniques to achieve better trade-offs between model size and performance.

Ongoing research efforts are focused on developing hybrid pruning strategies that combine the strengths of different pruning methods. These approaches aim to strike a balance between aggressive model compression and minimal performance degradation, paving the way for the widespread implementation of pruned models in various edge-computing applications.

#### 4.1.3. Knowledge Distillation

Knowledge distillation involves transferring knowledge from a large, cumbersome model (the teacher) to a smaller, more efficient model (the student). Techniques such as Hinton’s Knowledge Distillation [84] and Born-Again Networks [85] aid in this knowledge transfer. Recent advancements incorporate attention mechanisms and multi-stage distillation to enhance the student model performance and accuracy. Knowledge distillation is pivotal for transferring knowledge from large, complex models to smaller, efficient ones, facilitating deployment on resource-constrained edge-computing devices. Various approaches have evolved over time, enabling the compression of complex models without significant performance loss. Early techniques focused on transferring knowledge from a large, well-trained teacher model to a smaller student model, typically involving mimicking the teacher’s behaviour through soft labels or intermediate representations. Notable early approaches include the following:

- Hinton’s Knowledge Distillation: Proposed by Geoffrey Hinton in 2015, this pioneering technique involved training the student model to match the softened probabilities generated by the teacher model. It provided a foundational framework for subsequent developments in knowledge distillation, emphasizing the importance of transferring rich knowledge representations from complex models to compact ones.
- Born-Again Networks: Introduced in 2017, this approach focused on leveraging knowledge distillation to improve the performance of the teacher model itself. By training the teacher model on its own soft targets, the model’s performance was enhanced, leading to more effective knowledge transfer to the student model.

To address the limitations of early knowledge distillation methods and improve the efficiency of knowledge transfer, researchers have developed advanced techniques, including the following:

- Attention Mechanism-Based Distillation: These approaches incorporate attention mechanisms to guide the student model in focusing on crucial details provided by the teacher model. Attention-based distillation techniques facilitate the transfer of intricate knowledge representations, enabling the student model to capture important patterns and nuances present in the data [86].
- Multi-Stage Distillation: Multi-stage distillation techniques refine the knowledge transfer process iteratively, allowing the student model to learn from multiple stages of the teacher’s learning process. By progressively transferring knowledge across different stages, the student model can capture a more comprehensive understanding of the underlying data distribution, resulting in improved performance and robustness [87].

Recent advancements in knowledge distillation have focused on integrating self-distillation techniques and exploring the synergy between different distillation approaches. These developments aim to enhance the scalability and adaptability of knowledge distillation methods, enabling the efficient deployment of compact and accurate CV models in diverse edge-computing applications.

#### 4.1.4. Hardware-Aware Optimization

Hardware-aware optimization techniques adapt neural network models to efficiently utilize the unique hardware architecture of edge devices. Compiler optimizations, platform-specific libraries, and hardware-aware neural network design methods facilitate the creation of optimized models for mobile phones, IoT devices, and embedded systems. These techniques have evolved alongside advancements in edge-computing hardware and architectures to meet the specific demands of diverse edge-computing platforms. Hardware-aware optimization is crucial for tailoring and optimizing deep learning models to leverage particular hardware architectures and constraints in edge-computing environments. Various approaches have emerged over time, enabling the development of efficient CV models suited to various edge-computing devices [88]. Initially, hardware-aware optimization techniques focused on exploiting platform-specific libraries and compiler optimizations to enhance the performance and energy efficiency of deep learning models on edge devices. These methods prioritized the efficient utilization of hardware features like vectorized operations and specialized accelerators to speed up inference and reduce computational overhead. As the demand for deploying complex CV models on resource-constrained edge devices grew, researchers developed advanced hardware-aware optimization strategies, including the following:

- Neural Network Design for Specific Hardware: These approaches involve designing neural network architectures tailored to the the specific hardware characteristics of edge devices. By customizing the network structure to exploit hardware features such as parallel processing capabilities and memory hierarchies, these techniques optimize the overall performance and energy efficiency of models deployed on edge devices [89].
- Compiler Optimizations for Edge Devices: Compiler optimization techniques have been developed to transform high-level deep learning model representations into efficient executable code optimized for specific edge-computing platforms. These optimizations include code transformations and scheduling techniques that leverage the underlying hardware architecture to improve the performance and energy efficiency of deployed models [90].

Recent advancements in hardware-aware optimization have focused on integrating automated optimization tools and exploring the synergy between hardware-aware design and model-specific optimizations. These developments aim to facilitate the seamless integration of deep learning models with diverse edge-computing platforms, enabling efficient inference and analysis while minimizing resource utilization and energy consumption. Challenges still exist in achieving optimal performance and energy efficiency while ensuring compatibility with a wide range of edge-computing devices. Ongoing research efforts are addressing these challenges by developing adaptive optimization techniques that dynamically adjust model configurations based on real-time hardware constraints and performance requirements, thereby enabling the deployment of highly efficient CV models in edge-computing environments.

#### 4.1.5. Federated Learning

Federated learning enables model training across decentralized edge devices while safeguarding data privacy. Techniques like secure aggregation, differential privacy, and encryption protocols ensure the confidentiality of sensitive data. Federated averaging and secure communication protocols support collaborative model training and knowledge sharing without compromising privacy. This method gained traction with the introduction of secure aggregation and differential privacy. Ongoing research aims to improve the security, efficiency, and scalability of federated learning for edge computing. It has become a potent tool for collaborative model training across distributed edge devices while upholding privacy and security. Various federated learning approaches have evolved to tackle challenges in efficient and secure collaborative learning in distributed edge environments. Early techniques emphasized secure aggregation and differential privacy to maintain data privacy during training. These methods laid the groundwork for secure and efficient collaborative learning across multiple edge devices without centralized data sharing [91]. Advanced strategies include the following:

- Efficient Communication Protocols: These approaches aim to minimize communication overhead and optimize the exchange of model updates and gradients across distributed edge devices. By implementing efficient communication protocols, such as adaptive quantization and compression techniques, federated learning systems can achieve faster convergence and reduced communication costs while maintaining data privacy and security.
- Enhanced Security Protocols: Advanced federated learning systems integrate robust security protocols, including Secure Multiparty Computation (SMC) and homomorphic encryption, to protect sensitive data and model parameters during collaborative model training. These protocols ensure that privacy-sensitive information remains secure and encrypted throughout the entire learning process, enabling decentralized edge devices to participate in collaborative learning without compromising data privacy.

Recent advancements in federated learning have focused on optimizing communication efficiency, enhancing security protocols, and improving model aggregation techniques. These developments aim to address the scalability and reliability challenges of federated learning systems, enabling the deployment of efficient and collaborative CV models in diverse edge-computing environments. Challenges persist in achieving seamless coordination and synchronization across distributed edge devices while maintaining data privacy and security. Ongoing research efforts are addressing these challenges by exploring advanced encryption techniques, federated optimization algorithms, and adaptive communication strategies, paving the way for the widespread implementation of federated learning in various edge-computing applications.

#### 4.1.6. Model Compression

Model compression techniques aim to decrease the size of deep learning models without sacrificing performance. Common methods include weight sharing, low-rank factorization, and parameter regularization. Recent advancements like dynamic network surgery dynamically prunes and grows networks during training, striking a balance between model size and performance. Significant developments such as dynamic network surgery and adaptive compression strategies emerged to tackle challenges related to deploying deep learning models on resource-constrained edge devices. Model compression is crucial for reducing the size and complexity of deep learning models, enabling efficient deployment on edge-computing devices. Various approaches have evolved over time, focusing on minimizing model size while maintaining accuracy and performance. Early techniques concentrated on reducing parameters and operations through methods like weight sharing and low-rank factorization, aiming to cut memory requirements and computational overhead without compromising performance. Advanced strategies include the following:

- Parameter Regularization Techniques: These techniques aim to impose constraints on model parameters during training to prevent overfitting and reduce the model’s complexity. Methods such as L1 and L2 regularization encourage sparsity in the weight matrices, leading to more compact models with improved generalization capabilities and reduced memory footprint [92].
- Dynamic Network Surgery: Dynamic network surgery techniques dynamically adjust the network architecture during the training process, allowing the model to grow or shrink based on the task requirements. By adaptively adding or removing network components, dynamic network surgery enables the creation of highly efficient and task-specific models tailored for edge-computing applications [93].

Recent advancements in model compression have focused on integrating sparsity-inducing techniques, knowledge distillation, and hybrid compression methods. These developments aim to strike a balance between aggressive model compression and minimal performance degradation, paving the way for the widespread implementation of compressed models in various edge-computing applications. Challenges persist in achieving significant model compression while maintaining the robustness and interpretability of CV models. Ongoing research efforts are addressing these challenges by exploring advanced compression algorithms, hybrid model architectures, and adaptive compression strategies, enabling the deployment of lightweight and efficient CV models in edge-computing environments.

### 4.2. Computer Vision (CV) Packages and Libraries

To facilitate the development of CV models and applications, a range of software packages and libraries have been introduced including TensorFlow v2.16.1, PyTorch v2.4, OpenCV v4.10.0, Convolutional Architecture for Fast Feature Embedding (Caffe) v1.0, scikit-image v0.24.0, and SimpleCV v1.3s. A brief detail of each of these packages is provided as follows:

- TensorFlow is a widely adopted end-to-end open-source platform introduced by the Google Brain’s Machine Intelligence team for CV and neural network development, offering a comprehensive and flexible ecosystem encompassing various tools, libraries, and community resources [94]. TensorFlow provides stable Python and C++ APIs, alongside a non-guaranteed backward compatible API for other programming languages.
- PyTorch, initially developed by Meta AI, has emerged as one of the leading open-source ML libraries and serves as a versatile tool for CV development [95]. Offering a polished Python interface and a secondary C++ interface, PyTorch caters to diverse user preferences and development requirements. PyTorch supports ONNX for seamless model conversion between frameworks. PyTorch’s Tensor class facilitates efficient storage and manipulation of multi-dimensional arrays, offering seamless integration with CUDA-capable GPUs and ongoing support for diverse GPU platforms.
- OpenCV is an open-source CV library which serves as a foundational framework for a wide array of CV applications, offering accessibility and adaptability through its Apache 2 licensing [96]. Featuring over 2500 optimized algorithms, OpenCV enables users to tackle diverse CV tasks (e.g., face detection, optical tracking, and object detection). OpenCV is one of most widely adopted CV packages with around 47 thousand robust users. It supports multiple programming languages including C++, Python, Java, and MATLAB, as well as major OSs such as Windows, Linux, Android, and macOS, ensuring its broad accessibility and integration. Ongoing development efforts are directed towards enhancing GPU acceleration through CUDA and OpenCL interfaces, ensuring that OpenCV remains at the forefront of innovation in CV research and application development.
- Caffe is one of the initially developed deep learning frameworks by Berkeley AI Research (BAIR) designed with a focus on expression, speed, and modularity [97]. Caffe operates under the BSD 2-Clause license, fostering an open and collaborative development environment. Caffe offers seamless switching between CPU and GPU for training and deployment, facilitating versatility across various computing environments, from high-performance GPU machines to commodity clusters and mobile devices. Caffe project, at the time of its launch, had over 1000 members contributing to enhance its capabilities; however, it could not keep the pace as other libraries like PyTorch and TensorFlow took over. Although Caffe is not actively maintained any more, there is still support from NVIDIA to run Caffe on the latest generation of GPUs using cuDNN Caffe library. Caffe is still popular among the community; however, Caffe2 is actively adopted by researchers as the successor.
- Scikit-image, developed by Stéfan van der Walt and formerly known as scikits.image, is a Python-based open-source library dedicated to image-processing tasks. It features a comprehensive suite of algorithms covering segmentation, geometric transformations, colour space manipulation, and feature detection [98]. Designed to seamlessly integrate with Python’s numerical and scientific libraries, such as NumPy and SciPy, scikit-image offers a robust ecosystem for image analysis and manipulation. Leveraging a predominantly Python-based architecture, scikit-image optimizes performance by implementing core algorithms in Cython, striking a balance between ease of use and computational efficiency.
- SimpleCV is an accessible framework designed for the development of open-source CV leveraging the capabilities of OpenCV and the simplicity of the Python programming language [99]. SimpleCV aims to cater to both novice and experienced programmers, providing a comprehensive platform for basic CV functions as well as an elegant programming interface for advanced users. With features for the easy extraction, sorting, and filtering of image information, as well as fast manipulations with intuitive naming conventions, SimpleCV streamlines the process of developing CV applications. Moreover, it abstracts away the complexities of underlying CV libraries, such as OpenCV, allowing users to focus on application development without the need to delve into technical details like bit depths, file formats, or linear algebra concepts. In essence, SimpleCV enables users to harness the power of CV without unnecessary barriers, making the field more accessible and approachable for all.

#### Comparative Analysis

Each of the above-mentioned CV development packages offers unique strengths to meet various needs within the research and development communities. In terms of modularity and flexibility, OpenCV, PyTorch, and TensorFlow stand out. OpenCV provides a vast collection of modular components, enabling the development of complex applications with its extensive library of algorithms. PyTorch and TensorFlow, designed for deep learning and neural networks, offer modular architectures that support rapid prototyping and custom module integration. Although Caffe is not actively maintained, it still provides modularity through configuration files for model definition and optimization. Scikit-image and SimpleCV offer simpler modular structures that are easy to use and integrate.

Regarding performance and scalability, TensorFlow and PyTorch lead the way with support for GPU acceleration, mixed precision training, and large-scale deployments. They are optimized for high performance and can efficiently scale across multiple GPUs and nodes. OpenCV also optimizes performance through hardware acceleration (MMX and SSE instructions) and is enhancing GPU acceleration through CUDA and OpenCL. Caffe remains relevant with its seamless CPU–GPU switching, facilitating efficient deployment across diverse computing environments. SimpleCV, while user friendly, may not match the performance and scalability of the more advanced frameworks. Scikit-image focuses on efficiency with Cython-implemented core algorithms, making it suitable for real-time processing on less powerful hardware.

In terms of ease of use and accessibility, SimpleCV and scikit-image are particularly noted for their user-friendly interfaces, catering to users with limited expertise. They simplify complex tasks, making them suitable for beginners and rapid prototyping. TensorFlow, with its stable Python and C++ APIs, and PyTorch, known for its intuitive Python interface, balance ease of use with advanced functionality, making them accessible to a broad spectrum of users. OpenCV, while powerful, can have a steeper learning curve due to its extensive functionalities. Caffe, despite its initial complexity, simplifies model definition through configuration files, easing the learning process somewhat.

All these packages benefit from strong community support. TensorFlow and PyTorch, backed by Google and Meta, respectively, have vibrant communities that contribute to continuous development and innovation. OpenCV, with its widespread adoption, has a robust community of over 47 thousand users. Caffe, although less active now, still enjoys support from NVIDIA for running on the latest GPUs. Scikit-image and SimpleCV have dedicated user bases and are actively maintained through community-driven development, ensuring they remain useful and relevant.

In terms of deployment capabilities, TensorFlow and PyTorch excel with support for ONNX export and efficient scaling across various platforms. OpenCV’s focus on real-time vision tasks and optimization techniques ensure its suitability for deployment in diverse applications. Caffe’s seamless CPU–GPU switching facilitates versatile deployment environments and, despite being less prominent now, it still offers valuable deployment options, especially with NVIDIA’s support. SimpleCV simplifies deployment by abstracting complex details, making it accessible for quick application development. Scikit-image, integrated with the Python scientific stack, allows easy deployment for image-processing tasks within broader scientific workflows. Table 4 presents the comparison to CV development libraries in terms of license, optimization language, and application.

### 4.3. Computer Vision (CV) Development Frameworks

Several frameworks have emerged in the CV domain over the years in regard to the development (e.g., model training, fine-tuning, and exporting) of models for various CV application tasks including classification, detection, segmentation, and tracking. A few of the highlighted frameworks adopted by the research community include Detectron2 v0.6, NVIDIA TAO Toolkit v5.3.0, OpenMMLab, and Ultralytics v8.2.64. A brief introduction to each of the framework is listed as follows:

- Detectron2, developed by Facebook AI Research (FAIR) in 2018 [100], is a widely adopted open-source framework among the research community and offers a range of detection and segmentation model variants. It is built on PyTorch and known for its modularity, flexibility, and performance. Detectron2 model zoo includes a wide array of the latest object detection and instance segmentation model variants of Faster R-CNN. Furthermore, it uses GPU acceleration with mixed precision training enabling it to achieve higher inference speeds. In addition, it simplifies the deployment of models to production by offering standard training workflows and model conversion capabilities.
- The NVIDIA TAO Toolkit, unveiled in 2020 by NVIDIA, emerges as a leading solution designed for CV and AI applications, particularly suited for edge computing and embedded systems [101]. Developed to streamline AI model training and deployment, TAO simplifies the intricacies of deep learning frameworks like TensorFlow and PyTorch, offering a low-code approach to model customization and optimization. Leveraging pre-trained vision AI models from the NVIDIA GPU Cloud (NGC), users can fine-tune and customize models effortlessly, culminating in trained models deployable across a spectrum of platforms, from GPUs to CPUs and MCUs. Key features of the TAO Toolkit include its AutoML capability, which streamlines model training by automating hyperparameter tuning, and its support for model pruning and quantization-aware training, optimizing model size and inference performance. Additionally, TAO facilitates seamless deployment on various devices through its support for ONNX export and multi-GPU/multi-node training, ensuring scalability and efficiency.
- OpenMMLab, introduced in October 2018, is a comprehensive and modular open-source platform for the development of deep learning-driven CV applications [102]. Built upon the PyTorch framework, OpenMMLab leverages MMEngine to provide a universal training and evaluation engine, alongside MMCV, which offers essential neural network operators and data transforms. With over 30 vision libraries, 300 implemented algorithms, and a repository containing over 2000 pre-trained models, OpenMMLab continues to drive innovation and empower developers with the tools needed to tackle complex CV tasks effectively.
- Ultralytics platform, released in 2019, has gained fairly rapid attention in recent years for its YOLO series of models in object detection and instance segmentation research [103]. Ultralytics’ open-source projects on GitHub provide state-of-the-art solutions for a diverse range of AI tasks, spanning detection, segmentation, classification, tracking, and pose estimation. With an open and inclusive approach, Ultralytics actively seeks feedback, feature requests, and bug reports from its user base, ensuring continuous improvement and innovation. Leveraging the power of PyTorch and a range of compatible models, Ultralytics empowers users with features such as mixed precision training, real-time model evaluation, and visualization, facilitating a seamless transition from model creation to practical deployment.

#### Comparative Analysis

Each of the above-mentioned CV development frameworks offers unique strengths addressing the needs of the research community. This section provides the subjective comparison of the development frameworks in terms of modularity, performance, ease of use, community support, and deployment capabilities.

In terms of modularity and flexibility, Detectron2 and OpenMMLab are better in providing modular and extensible designs. Built on PyTorch, these frameworks allow for the rapid prototyping and integration of custom modules, which is particularly beneficial for research and complex applications. Detectron2’s architecture supports a clean separation of components, facilitating experimentation and innovation. Similarly, OpenMMLab offers a comprehensive ecosystem, making use of MMEngine and MMCV to provide a universal training and evaluation engine. This modularity, however, comes at the cost of a steeper learning curve, making these frameworks more challenging for beginners.

In the context of performance and scalability, Detectron2 leverages GPU acceleration and mixed precision training, offering superior speed and scalability, making it suitable for both research and production environments. NVIDIA TAO Toolkit, on the other hand, emphasizes efficiency through its support for multi-GPU and multi-node training. By utilizing pre-trained models from the NGC and supporting ONNX export, TAO Toolkit ensures scalable and efficient deployment across various platforms. However, it is primarily optimized for NVIDIA hardware, potentially limiting its use for those using other platforms. While Ultralytics focuses on making AI models accessible and easy to deploy, it may face limitations in scalability and performance compared to frameworks specifically designed for large-scale deployments.

On the subject of ease of use and accessibility, the low-code approach of NVIDIA TAO Toolkit simplifies the complexities of deep learning frameworks like TensorFlow and PyTorch, making it accessible to users with limited expertise. This approach streamlines model training and customization, empowering developers to harness the power of transfer learning with minimal data and coding effort. Ultralytics also prioritizes accessibility, providing state-of-the-art solutions that are efficient to train and effortless to deploy. It supports features like mixed precision training and real-time model evaluation, facilitating a seamless transition from model creation to practical deployment. In contrast, the complexity and resource demands of Detectron2 and OpenMMLab might be a hurdle for beginners or those with limited computational resources.

In terms of community support and collaboration, Detectron2 benefits from strong community support due to its foundation in PyTorch and backing by FAIR. OpenMMLab fosters collaboration between academia and industry, bridging the gap between theoretical research and practical applications. With over 30 vision libraries and 300 implemented algorithms, supported by a robust repository of over 2000 pre-trained models, OpenMMLab drives innovation and empowers developers. Ultralytics engages a vibrant community of contributors, ensuring regular updates and active community support. It welcomes feedback, feature requests, and bug reports from its user base, fostering continuous improvement and innovation. NVIDIA TAO Toolkit is supported by NVIDIA’s extensive resources and infrastructure, ensuring robust technical support and updates.

Finally, in the context of deployment capabilities, NVIDIA TAO Toolkit leads in deployment capabilities, supporting ONNX export, and ensuring scalability and efficiency across various devices, from GPUs to CPUs and MCUs. Detectron2 also simplifies the deployment of advanced models to production, offering standard training workflows and model conversion capabilities for cloud and mobile deployment. OpenMMLab provides efficient toolchains targeting diverse backends and devices, streamlining the deployment process. Ultralytics focuses on practical deployment, making state-of-the-art AI models accessible and easy to deploy, although it may not offer the same level of advanced features and scalability as more specialized frameworks. Table 5 presents a comparison of the CV development frameworks.

### 4.4. Computer Vision (CV) Packages for Hardware Deployment

The deployment of trained CV models to production on SBCs requires optimization to achieve a high performance on resource-constrained hardware. There exist a variety of packages to deploy the models on hardware including PyTorch Mobile, OpenVINO v2024.2, ONNX v1.16.1, TensorRT v8.6.0, and Tensor Flow Lite v2.13. A brief introduction to each of the packages is listed as follows:

- PyTorch Mobile is a leading solution for deploying ML models on low-power mobile and edge-computing devices, designed for compactness and performance in CV tasks [104]. It is part of the PyTorch ecosystem allowing a smooth transition from model training to deployment. Some highlighted features include the privacy preserving federating learning capability, cross-platform support, support for TorchScript, and integration with optimization techniques.
- OpenVINO, short for Open Visual Inference and Neural network Optimization, is an open-source toolkit developed by Intel for optimizing and deploying AI inference on a variety of devices, particularly embedded systems and edge-computing devices [105]. By leveraging models trained on popular frameworks like TensorFlow and PyTorch, OpenVINO enables users to optimize the model inference on low-power and resource-constrained SBCs. The core components of the OpenVINO toolkit encompass the OpenVINO Model Converter (OVC), OpenVINO Runtime, and a versatile set of plugins catering to CPUs, GPUs, and heterogeneous computing environments. Additionally, the toolkit provides a suite of samples and frontends, facilitating model conversion, inference, and transformation tasks with ease.
- ONNX is an open-source initiative towards a unified solution for the seamless interoperability of models across various development frameworks [106]. ONNX enables AI developers to transcend framework limitations and effortlessly exchange models between platforms such as TensorFlow, PyTorch, and Caffe. It facilitates access to hardware accelerators through compatible runtimes and libraries, optimizing performance across a spectrum of hardware configurations.
- TensorRT, introduced by NVIDIA, is a high-performance deep learning inference engine designed for the efficient deployment of models on edge devices and low-power systems [107]. Compatible with popular frameworks like TensorFlow and PyTorch, TensorRT offers a suite of optimization techniques aimed at minimizing memory usage and computational overhead. Leveraging the NVIDIA CUDA parallel programming model, TensorRT enables developers to enhance inference performance through quantization, layer fusion, kernel tuning, and other optimization methods on NVIDIA GPUs. Furthermore, it supports INT8 quantization-aware training, post-training quantization, and FP16 optimizations.
- TensorFlow Lite is a specialized version of TensorFlow explicitly designed for deployment on edge-computing and embedded devices [108]. By optimizing for on-device operations, TensorFlow Lite addresses crucial constraints such as latency, privacy, connectivity, size, and power consumption, making it ideal for edge-computing scenarios where real-time processing and data privacy are significant. With hardware acceleration and model optimization techniques, TensorFlow Lite delivers high-performance inference, ensuring the efficient execution of ML models on resource-constrained devices.

#### Comparative Analysis

In the context of deploying ML models on mobile and edge devices, this section compares the above-mentioned hardware deployment packages in terms of compatibility, performance, deployment ease, hardware optimization, and community and industry adoption.

In terms of compatibility and interoperability, ONNX stands out for its ability to facilitate seamless interoperability among various deep learning frameworks. By providing a standard format for representing ML models, ONNX allows developers to transition models between TensorFlow, PyTorch, and other platforms. This feature is crucial for projects that require flexibility across different development frameworks. PyTorch Mobile and TensorFlow Lite, while primarily integrated with their respective ecosystems, also support interoperability through ONNX. OpenVINO, with its model converter, supports models trained on TensorFlow and PyTorch, enhancing its compatibility with various frameworks. The TensorRT on the other hand is suitable for optimized inferencing on the NVIDIA hardware and part of NVIDIA’s end-to-end AI development eco-system.

On the subject of performance and optimization, TensorRT leads in high-performance deep learning inference, leveraging NVIDIA’s CUDA parallel programming model and optimization techniques like quantization and kernel tuning to achieve significant speedups. TensorFlow Lite also focuses on optimizing performance for edge devices, supporting hardware acceleration and model optimization techniques. OpenVINO provides robust optimization for low-power devices, utilizing Intel’s hardware capabilities. PyTorch Mobile aims for compactness and performance, but its optimization may not be as advanced as TensorRT or OpenVINO.

In the context of deployment ease and workflow, TensorFlow Lite and PyTorch Mobile offer streamlined workflows for transitioning from model training to deployment within their ecosystems. TensorFlow Lite’s broad platform support and comprehensive APIs simplify deployment across various devices. PyTorch Mobile provides a similar streamlined experience within the PyTorch ecosystem, emphasizing ease of use. OpenVINO, while powerful, requires a more detailed understanding of its components and may present a steeper learning curve. TensorRT, with its advanced optimization features, also demands familiarity with NVIDIA’s ecosystem, potentially posing challenges for less experienced developers.

In regard to hardware optimization, TensorRT is highly optimized for NVIDIA GPUs, enabling enhanced performance through techniques like INT8 quantization and FP16 optimizations. OpenVINO supports a wide range of hardware devices, including Intel CPUs, ARM CPUs, and Intel GPUs, offering flexibility and scalability. TensorFlow Lite provides hardware acceleration for various devices, including microcontrollers and smartphones. PyTorch Mobile, while optimized for mobile devices, may not offer the same level of hardware-specific optimizations as TensorRT or OpenVINO.

In terms of community and industry adoption, TensorFlow Lite and PyTorch Mobile benefit from strong community support and comprehensive documentation, driven by the popularity of the TensorFlow and PyTorch platforms. OpenVINO, backed by Intel, enjoys robust support within the industry, particularly for edge and embedded applications. TensorRT, supported by NVIDIA, is widely adopted for high-performance inference tasks, especially in applications requiring low latency. ONNX, as an open ecosystem, continues to evolve with widespread support across frameworks and hardware, fostering innovation and collaboration within the AI community. Overall, all of the packages have good community support and adoption based on the specific target application. On the subject of privacy and security, PyTorch Mobile and TensorFlow Lite address these concerns by enabling on-device ML, reducing the need to transmit data to external servers. PyTorch Mobile’s support for federated learning methodologies further enhances privacy-preserving functionalities. OpenVINO and TensorRT also support on-device inference, which can help mitigate the privacy risks associated with cloud-based processing. Table 6 presents the comparison of the CV packages for hardware deployment.

**Figure 2:**
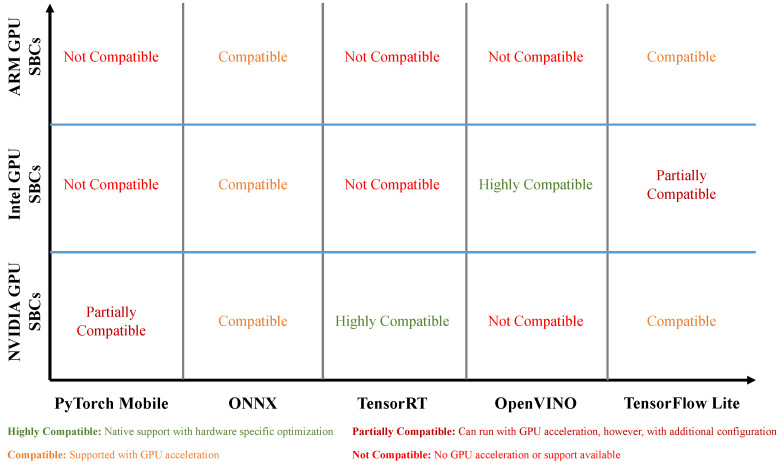
Cross-platform mapping of CV hardware deployment package against Single-Board Computers (SBCs).

It is essential to examine the cross-platform compatibility of these packages specifically for SBCs equipped with different types of GPUs. This analysis will assist developers in selecting the appropriate packages for specific SBCs, as not all packages support various GPU types uniformly. Figure 2 illustrates the mapping of packages across three different GPU-based SBCs: those with NVIDIA GPUs, Intel GPUs, and ARM GPUs.

From this mapping, it is evident that NVIDIA GPU-based SBCs offer native, highly optimized support for TensorRT, making it the recommended package for high-performance inference. These SBCs also support ONNX Runtime and TensorFlow Lite with GPU acceleration, though the inference performance is not as optimized. PyTorch Mobile is partially supported on NVIDIA GPUs, requiring additional configuration and driver installation for GPU acceleration. However, OpenVINO is not supported on NVIDIA GPU-based SBCs.

In the case of Intel GPU-based SBCs, the OpenVINO package is natively supported with hardware-specific optimizations, thus recommended for achieving maximum performance. ONNX Runtime is supported with GPU acceleration, while TensorFlow Lite is partially supported. Conversely, TensorRT and PyTorch Mobile are not compatible with Intel GPU-based SBCs.

For ARM GPU-based SBCs, both the ONNX and TensorFlow Lite packages are supported for GPU-accelerated inference, making them the recommended choices for development. The PyTorch Mobile, TensorRT, and OpenVINO packages are not compatible with ARM GPU-based SBCs. It is important to note that, while PyTorch Mobile may not be compatible, the full PyTorch package can be configured with GPU support.

## 5. Challenges, Limitations, and Opportunities

As the integration of CV technology into SBCs continues to grow, a number of limitations and challenges must be addressed to fully realize its potential. The SBCs face significant constraints in computational power, memory, and real-time processing capabilities. Overcoming these challenges is essential for deploying efficient, robust, and scalable CV solutions. Conversely, ongoing advancements and future opportunities in edge-computing hardware, optimized algorithms, and AI accelerators hold promise for enhancing the performance and applicability of CV models in diverse environments. This section delves into the current limitations and challenges faced by CV implementations on SBCs, followed by an exploration of future opportunities that could drive innovation and improvement in this field.

### 5.1. Current Limitations and Challenges

- Limited Computational Power: SBCs have restricted computational capabilities and necessitate highly optimized models that balance computational complexity and performance to maintain real-time processing capabilities. Techniques such as model pruning, quantization, and knowledge distillation are essential to reduce the model’s computational footprint while preserving accuracy. Additionally, optimizing the computational graph and leveraging specialized instruction sets at CUDA level can further enhance inference performance.
- Memory and Storage Constraints: SBCs often feature limited RAM and non-volatile storage, which restricts the size and complexity of deployable models. This limitation requires the deployment of compact neural network architectures (e.g., MobileNets or SqueezeNet) and efficient memory management strategies to fit models within the available memory without significantly sacrificing performance. Memory mapping techniques and in-memory computation strategies are critical for maximizing the usage of available resources.
- Real-Time Processing: Real-time video analytics on SBCs demands efficient processing pipelines capable of handling high-resolution video feeds and complex CV tasks with minimal latency. Techniques like pipeline parallelism, edge-computing strategies, and the use of lightweight neural networks (e.g., YOLO-lite or Tiny YOLO) are crucial for achieving the desired throughput and response times. Implementing hardware-level model optimization and leveraging asynchronous processing can significantly reduce processing times and enhance real-time capabilities.
- Maintenance and Management: Deploying and managing CV models on distributed edge devices involve complex challenges related to monitoring, updates, and maintenance. This necessitates the implementation of Over-The-Air (OTA) updates, remote diagnostics, and automated monitoring systems to ensure continuous operation and adaptability to evolving requirements. Utilizing containerization (e.g., Docker or Kubernetes) and employing Continuous Integration/Continuous Deployment (CI/CD) pipelines can streamline the deployment and maintenance processes.
- Heterogeneity in Edge Devices: The diversity of edge devices, each with distinct hardware capabilities, OS, and software ecosystems, present significant compatibility challenges. Developing universally compatible CV models requires using cross-platform development frameworks, and leveraging hardware abstraction layers to ensure consistent performance across different devices. Implementing device-specific optimizations and leveraging cloud-based orchestration can further enhance compatibility and performance.
- Heat Management in SBCs: SBCs often rely on passive cooling mechanisms, which may be insufficient for systems running 24/7. Continuous operation under high computational loads can lead to overheating, resulting in thermal throttling or system failures. Even active cooling solutions may not provide adequate heat dissipation, necessitating the design of efficient thermal management strategies, such as heat sinks, heat pipes, and advanced cooling systems, to ensure reliable long-term operation.
- Power Consumption: SBCs designed for remote applications must contend with high power consumption, which can make them unsuitable for scenarios where energy efficiency is critical. Optimizing power usage through efficient hardware design, low-power components, and dynamic power-management techniques is essential to extend operational uptime and reduce overall energy costs. The integration of energy-efficient processors and peripherals, coupled with software optimizations for minimal power consumption during idle and active states, is crucial for enhancing the practicality of SBCs in remote deployments.

### 5.2. Future Opportunities

- Advancements in Edge-Computing Hardware: The development of next-generation embedded hardware, featuring more powerful processors, enhanced memory, and efficient energy utilization, will facilitate the deployment of more sophisticated CV models. Innovations in the GPU architectures and dedicated AI accelerators will further enhance the performance of SBC.
- Optimized Deep Learning Algorithms: The development of light-weight and yet efficient CV models specifically for mobile deployment is an active area of research. In addition, advancements in model optimization and quantization techniques will enable high-performance inference on SBCs.
- Integration of AI Accelerators: Embedding dedicated AI accelerators, such as Google’s Edge TPU, Intel’s Movidius, and NVIDIA’s Tensor cores, into edge devices will dramatically enhance inference speed and energy efficiency, enabling the real-time processing of more complex CV tasks directly on the edge. These accelerators provide specialized hardware designed to accelerate common deep learning operations (e.g., matrix multiplications and convolutions) and support parallel processing, significantly boosting performance. Elevated performances have already been observed for the latest NVIDIA Jetson boards with dedicated Tensor cores.
- Offline Functionality: Enhancements in edge-computing capabilities will allow CV systems to perform critical tasks offline, ensuring continuous operation even in the absence of stable network connectivity. This is particularly advantageous for applications in remote areas or environments with unreliable internet access, where uninterrupted real-time processing is essential. Developing robust fallback mechanisms and local data-storage solutions will support seamless offline functionality.
- Intelligent Power Management: Power management in SBCs stands out as a critical challenge that requires immediate attention to extend operational times effectively. Future research directions are increasingly focusing on AI-oriented approaches to power management and optimization at the device level. These approaches aim to leverage AI techniques such as ML and reinforcement learning to dynamically adjust power consumption based on workload demands and environmental conditions. Optimizing at the device level involves developing energy-efficient hardware designs and implementing intelligent power-saving algorithms.

## 6. Conclusions

In the field of SBCs, NVIDIA, ASUS, and Libre have emerged as the primary manufacturers, with NVIDIA leading the pack due to its advancements in GPU architectures. Comparative analysis across various performance-oriented specifications suggests that all the SBCs except NVIDIA Jetson TX2, NVIDIA Jetson NX, and NVIDIA Jetson Orin AGX are only suitable for entry-level CV tasks because of limited memory, old generation GPUs, and inefficient cooling mechanisms. Although the latest SBCs from the NVIDIA Jetson series (i.e., NVIDIA Jetson Orin Nano, NVIDIA Jetson Orin AGX, and NVIDIA Jetson Xavier NX) comes with the latest generation of GPU architectures and dedicated Tensor cores for AI tasks, they come at a higher price value (e.g., entry-level NVIDIA Jetson Orin Nano at USD 800). The deployment of CV models on SBCs faces challenges, including limited computational resources and real-time processing demands. To address these challenges, various model-level optimization techniques such as model quantization, compression, pruning, knowledge distillation, and federated learning have been explored. Development libraries such as TensorFlow, PyTorch, Caffe, OpenCV, scikit-image, and Simple CV have gained prominence, with TensorFlow and PyTorch emerging as clear leaders. PyTorch holds a slight advantage due to its customization capabilities and ease of development at the core level. For development frameworks, Detectron2, TAO Toolkit, OpenMMLab, and Ultralytics have risen to prominence for training and exporting CV models. OpenMMLab leads in terms of the availability of the latest models and diverse CV tasks, although it may require additional optimization for deployment on leading NVIDIA Jetson boards. Conversely, TAO Toolkit offers a range of pre-built and tested export models, facilitating seamless integration with NVIDIA Jetson boards for optimal performance. Several packages have been specifically tailored for exporting models for SBC deployment, including PyTorch Mobile, OpenVINO, ONNX, TensorRT, and TensorFlow Lite. Among these, TensorRT stands out within the NVIDIA ecosystem, delivering superior performance on Jetson boards compared to other alternatives. The cross-platform mapping of hardware deployment packages against the SBCs reveals that TensorRT with NVIDIA GPU-based SBCs and OpenVINO with Intel GPU-based SBCs are highly compatible combinations with native hardware level support. ONNX, on the other hand, can run on all the SBCs with GPU support; however, the inference will not be highly optimized at the hardware scale. Limited computational power, memory constraints, lack of real-time processing capability, inefficient power management, and outdated heat management are some highlighted limitations of SBCs in the context of CV solutions. Future research directions include the development of innovative GPU architectures, hardware-specific optimized CV algorithms, integration of AI accelerators, improved heat management designs and AI-driven power-management strategies. In the context of the presented review, a detailed hardware scale benchmarking against the range of CV tasks in future will provide a more technical comparison of the available SBCs. 

## Figures and Tables

**Table 1:** Comparison of existing GPU-accelerated Single-Board Computers (SBCs).

	Name	Release Year	CPU Technology	GPU Technology	RAM	Storage	Avg Power Consumption	Dimensions (inch)	Price (USD)
ASUS	Tinker Board S	2021	Rockchip RK3288	ARM Mali-T764	2 GB DDR3	16 GB eMMC	≈5 W	3.37″ × 2.125″	USD 199
	Tinker Edge T	2019	NXP i.MX 8M	ARM Mali-T860	1 GB LRDDR4	8 GB eMMC	≈5–10 W	3.35″ × 2.20″	USD 240
	Tinker Board 2	2020	Rockchip RK3399	ARM Mali-T860	2 GB LPDDR4	16 GB eMMC	≈5–10 W	3.37″ × 2.12″	USD 120
	Tinker Board 3 N	2023	Rockchip RK3568	ARM Mali G52	4 GB LPDDR4	64 GB eMMC	≈5–10 W	4″ × 4″	USD 160
NVIDIA	Jetson Nano	2019	ARM Cortex-A57	128-core Maxwell	4 GB LPDDR4	External microSD	≈5–10 W	2.72″ × 1.77″	USD 249
	Jetson TX2	2017	ARM Cortex-A57	256-core Pascal	8 GB LPDDR4	32 GB eMMC	≈15 W	3.42″ × 1.96″	USD 810
	Jetson Xavier NX	2020	6-core Carmel	384-core Volta ^1^	8 GB LPDDR4	16 GB eMMC	≈10–30 W	2.74″ × 1.77″	USD 530
	Jetson AGX Orin	2023	ARM Cortex-A78AE	2048-core Ampere ^2^	32 GB LPDDR5	64 GB eMMC	≈15–60 W	4.33″ × 4.33″	USD 3000
	Jetson Orin Nano	2023	ARM Cortex-A78 AE	512-core Ampere ^3^	8 GB LPDDR5	External microSD	≈7–15 W	3.93″ × 3.11″	USD 800
Libre	Libre Tritium	2018	4 ARM Cortex-A7	ARM Mali-400	2 GB DDR3	External microSD	≈5 W	3.34″ × 2.20″	USD 35
	Libre Le Potato	2017	4 ARM Cortex-A53	ARM Mali-450	2 GB DDR3	External microSD	≈5 W	3.34″ × 2.20″	USD 30
	Libre Renegade	2018	4 ARM Cortex-A53	ARM Mali-450	4 GB DDR4	External microSD	≈5–10 W	3.34″ × 2.20″	USD 45
Others	VisionFive 2	2021	StarFive JH7110	BXE-4-32	8 GB LPDDR4	External microSD	≈10 W	3.93″ × 2.83″	USD 65
	ROCK PI N10	2021	ARM Cortex-A72	ARM Mali T860MP4	4 GB DDR3	8 GB eMMC	≈15–18 W	3.93″ × 3.93″	USD 199
	BeagleBone AI	2019	ARM Cortex-A15	PowerVR SGX544	1 GB	16 GB eMMC	≈5–10 W	3.50″ × 2.12″	USD 198
	HiKey970	2017	ARM Cortex-A7	ARM Mali-G72	6 GB LPDDR4	64 GB UFS	≈10–15 W	4.14″ × 3.93″	USD 239
	Coral Dev Board	2019	ARM Cortex-A53	GC7000 Lite Graphics	4 GB LPDDR4	8 GB eMMC	≈5 W	5.40″ × 3.90″	USD 200
	Coral Dev Mini	2020	ARM Cortex-A35	PowerVR GE8300	2 GB LPDDR3	8 GB eMMC	≈3 W	2.52″ × 1.89″	USD 100

^1^: 48 Tensor cores; ^2^: 64 Tensor cores; ^3^: 12 Tensor cores.

**Table 2:** Summary of a few use cases reported in literature where GPU-accelerated Single-Board Computers (SBCs) are deployed.

SBC	CV Task	Purpose	Model	Packages	Inference Performance	Reference
Jetson Nano	Detection	Pedestrian Detection	YOLOv5s	TensorRT	15 FPS	Chen and Wang [35]
	Classification	ImageNet Classification	MobileNetv2	TensorFlow, TensorRT, ONNX	0.020 s per image	Baller et al. [38]
	Classification	Binary Classification	MobileNetv3	PyTorch, ONNX, TensorRT	0.300 s per image	Swaminathan et al. [76]
	Detection	Ship Detection	YOLOv5	PyTorch with CUDA	4.86 FPS	Valencia et al. [40]
	Classification	Multiclass Custom	MobileNetv2	TensorRT	6 ms per image	Rosero-Montalvo et al. [77]
	Detection	Face Mask Detection	YOLOv4 Tiny	PyTorch with CUDA	13 FPS	Hakim et al. [41]
	Detection	Pedestrian Detection	SSD	PyTorch, TensorRT	8.7 FPS	Sarvajcz et al. [36]
	Classification	DeepFashion2 Classification	Custom CNN	CUDA, CuDNN	6.4 ms per image	Suzen et al. [42]
	Detection	COCO Detection	YOLOv3	PyTorch with CUDA	0.786 ms per image	Zhu et al. [43]
	Detection	Pedestrian Detection	YOLOv4	PyTorch with CUDA	8 FPS	Wang et al. [37]
	Classification	MNIST Classification	DarkNet	TensorFlow	0.217 ms per image	Tolmacheva et al. [39]
	Detection	Landing Platform Identification	YOLOv4 Tiny	TensorRT	36.48 FPS	Ma et al. [44]
	Dual Detection	Person and Weapon	SSD, YOLOv4	FP16 with TensorRT	1.4 FPS	Berardini et al. [45]
Jetson Nano	Detection	Plastic Bag Detection	YOLOv4 Tiny	TensortRT, FP32, DeepStream	16.4 FPS	Iqbal et al. [8]
	Detection	Ripe Coffee Detection	YOLOv3	TensorFlow	8180 ms per image	Beegam et al. [46]
Jetson TX2	Detection	PASCAL VOC Detection	SSD	PyTorch with CUDA Support	9.35 FPS	Chen et al. [47]
	Detection	COCO Detection	Custom CNN	OpenCV, TensorFlow	0.130 s per image	Taspinar and Selek [26]
	Classification	Multiclass Custom	MobileNetv2	TensorRT	3.1 ms per image	Rosero-Montalvo et al. [77]
	Classification	DeepFashion2 Classification	Custom CNN	CUDA, CuDNN	4.6 ms per image	Suzen et al. [42]
	Detection	Drone Detection	YOLOv3	PyTorch with CUDA	<1 FPS	Xun et al. [48]
	Detection	Vehicle Detection	YOLOv3 Tiny	TensorRT with FP16	18.3 FPS	Nguyen et al. [49]
	Detection	Pedestrian Detection	YOLOv3	PyTorch with CUDA	6.6 FPS	Afifi et al. [50]
	Detection	Person Detection	YOLOv8	PyTorch with CUDA	5.61 FPS	Byzkrovnyi et al. [51]
	Detection	Plastic Bag Detection	YOLOv4 Tiny	TensortRT, FP32, DeepStream	24.8 FPS	Iqbal et al. [8]
	Segmentation	Weed Segmentation	SegNet	Caffe, CUDA, cuDNN	0.56 s per image	Sa et al. [52]
	Detection	Pest Detection	YOLOv3 Tiny	PyTorch with CUDA	8.71 FPS	Chen et al. [53]
	Detection	Concrete Crack Detection	YOLOv3	OpenCV, PyTorch, CUDA	33 ms per image	Kumar et al. [54]
Jetson Xavier NX	Detection	Banana Ripeness Detection	YOLOv8 Nano	PyTorch with CUDA	13.9 ms per image	Aishwarya and Kumar [55]
	Detection	Face Mask Detection	YOLOv5s	PyTorch with CUDA	12 FPS	Aljaafreh et al. [56]
	Detection	COCO Detection	YOLOv4 Tiny	TensorRT with FP16	0.0035 ms per image	Shin and Kim [57]
	Detection	Vehicle and Pedestrian Detection	YOLOv8	DeepStream, TensorRT	6.7 ms per image	Wasule et al. [58]
	Detection	COCO Detection	YOLOv3	PyTorch with CUDA	0.252 ms per image	Zhu et al. [43]
	Detection	Pedestrian Detection	YOLOv4	PyTorch with CUDA	15 FPS	Wang et al. [37]
	Depth Estimation	Monocular Estimation	FastMDE	PyTorch, ONNX, TensorRT	30 ms per image	Dao et al. [60]
	Detection	Fire and Smoke Detection	YOLOv3	PyTorch, DeepStream	9.9 FPS	Chen et al. [59]
Jetson ORIN AGX	Classification	Tomato Disease Classification	MobileNetv2	ONNX with ONNXRuntime	0.4 ms per image	Zahid et al. [61]
	Detection	COCO Detection	SSD	TensorRT	48.41 FPS	Avila et al. [62]
	Detection	Ground Vehicle Detection	YOLOX	TensorRT with FP32	37 FPS	Bhattacharjee et al. [63]
	Detection	Surgical Instrument Detection	YOLOv5 Model	TensorRT with int8, DeepStream	2.3 ms per image	Belhaoua et al. [64]
	Detection+Tracking	Vehicle Detection	YOLOX	FP32 with TensorRT	87.2 FPS	Carvalho et al. [65]
Jetson ORIN Nano	Classification	Tomato Disease Classification	MobileNetv2	ONNX with ONNXRuntime	0.6 ms per image	Zahid et al. [61]
	Detection+Tracking	Vehicle Detection	YOLOX	FP32 with TensorRT	78.9 FPS	Carvalho et al. [65]
HiKey970	Detection	PASCAL VOC Detection	SSD	PyTorch with CUDA Support	1.45 FPS	Chen et al. [47]
	Classification	MNIST Classification	DarkNet	TensorFlow	0.460 ms per image	Tolmacheva et al. [39]
ASUS Tinker Board S	Detection	COCO Detection	Custom CNN	OpenCV, TensorFlow	0.245 s per image	Taspinar and Selek [26]
	Detection	Face Detection	Haar Features	OpenCV	0.2 s per image	Chen et al. [27]
	Classification	Face with Mask Classification	MobileNetv2	TensorFlow	Not reported	Jahan et al. [28]
ASUS Tinker Edge T	Classification	Pest Classification	ResNet 8	TFLite	3 ms per image	Tran and Tran [29]
Coral Dev Board	Classification	ImageNet Classification	MobileNetv2	TFLite, Quant Integer	0.004 s per image	Baller et al. [38]
	Segmentation	Eye Optical Disc Segmentation	UNet Model	TFLite	9 ms per image	Masot et al. [74]
	Classification	Multiclass Custom	MobileNetv2	TFLite with IQ quantization	2.9 ms	Rosero-Montalvo et al. [77]
	Classification	Tomato Disease Classification	MobileNetv2	ONNX with ONNXRuntime	1.16 ms per image	Zahid et al. [61]
	Detection	People Detection	Custom Model	OpenCV, TFLite	1 FPS	Petersson and Mohammedi [75]
	Detection	Person and Weapon	SSD, YOLOv4	int8 with TFLite	0.9 FPS	Berardini et al. [45]
BeagleBone AI	Classification	MNIST Classification	Custom Model	OpenCV, TFLite	0.524 ms per image	Bogacz and Qouneh [73]
VisionFive 2	Detection	Universal Object Detection	YOLOv3	OpenCV, PyTorch	427.70 ms per image	Jacob [72]

**Table 3:** Mapping of fundamental Computer Vision (CV) tasks to Single-Board Computers (SBCs).

CV Task	Model Complexity	Inference Speed	Visual Complexity	Single/Multiple Models	Input Streams	Recommended SBCs
Entry-Level ^1^ Classification	Simple (e.g., MobileNet)	Not Critical	Simple Background	Single Model Inference	Single Stream	Tinker Board S
						Tinker Board T
						Tinker Board 2
						Libre Tritium
						Libre Le Potato
						Libre Renegade
						ROCK PI N10
						BeagleBone AI
						Coral Dev Mini
Moderate-Performance ^2^ Classification	Moderate (e.g., ResNet50)	<1 FPS	Visually Challenging Background	Single Model Inference	Single Stream	Tinker Board 3N
						Jetson Nano
						VisionFive 2
						HiKey970
						Coral Dev Board
High-Performance ^3^ Detection and Tracking	Complex (e.g., YOLO, DINO)	>15 FPS	Complex and Dynamically Changing Background	Single Model Inference + Tracker	Single Stream	Jetson TX2
						Jetson Xavier NX
						Jetson Orin Nano
Very-High-Performance ^4^ Detection and Tracking	Complex (e.g., YOLO, DINO)	>15 FPS	Complex and Dynamically Changing Background	Multiple Model Inference + Tracker	Multiple Stream	Jetson AGX Orin

^1^: Develop an image-classification system to classify plant disease from plant leave images. ^2^: Develop an image-classification system to classify animal types from trap camera captured images. ^3^: Develop a video analytics system to detect and count vehicles in real time from live video feed. ^4^: Develop a video analytics system to detect, classify, and count vehicles in real time for multiple input streams.

**Table 4:** Comparison of Computer Vision (CV) libraries and packages.

Feature	OpenCV	TensorFlow	Caffe	PyTorch	Scikit-Image	SimpleCV
License	Apache 2	Apache 2	BSD 2-Clause	Custom (BSD-style)	BSD 3-Clause	BSD 3-Clause
Application	CV and ML	End-to-End ML	Deep Learning	ML and Deep Learning	Image Processing	Machine Vision
Language	C++, Python, Java, MATLAB	Python, C++, Java	C++, Python	Python, C++	Python	Python
Optimization	MMX, SSE	GPU (CUDA, OpenCL)	GPU Acceleration	GPU Acceleration	Cython	NA

**Table 5:** Comparison of Computer Vision (CV) development frameworks.

Feature	Detectron2	NVIDIA TAO Toolkit	OpenMMLab	Ultralytics
License	Apache 2	Proprietary	Apache 2	MIT
Developer	Facebook AI Research	NVIDIA	MMLAB	Ultralytics
Framework	PyTorch	TensorFlow, PyTorch	PyTorch	PyTorch
CV Tasks	Det, Seg	Det, Seg, Clas, Action, Pose	All	Det, Seg
AutoML	No	Yes	No	No
Export Options	TorchScript, ONNX	ONNX, TRT Engine	ONNX, Torch	ONNX, Torch

**Table 6:** Comparison of Computer Vision (CV) packages for hardware deployment.

Feature	PyTorch Mobile	OpenVINO	ONNX	TensorRT	TensorFlow Lite
License	BSD	Apache 2	Apache 2	NVIDIA License	Apache 2
Developer	Facebook AI Research	Intel	Linux Foundation	NVIDIA	Google
Framework Comparability	PyTorch	TensorFlow, PyTorch	TensorFlow, PyTorch, Caffe	TensorFlow, PyTorch, Caffe	TensorFlow
Optimization Techniques	Pruning, Quantization	Model Conversion	Framework Independent	Quantizations, Layer Fusion	Quantization, Operator Optimization
GPU Support	Upcoming	Yes (Intel GPUs)	Yes	Yes (NVIDIA GPUs)	Yes

## Data Availability

Data are contained within the article.

## Abbreviations

The following abbreviations are used in this manuscript:

CV	Computer Vision
GPU	Graphical Processing Unit
CNN	Convolutional Neural Network
SBC	Single-Board Computer
CUDA	Compute Unified Device Architecture
LiDAR	Light Detection and Ranging
AI	Artificial Intelligence
ARM	Advanced RISC Machine
CPU	Central Processing Unit
GPIO	General-Purpose Input/Output
DSI	Display Serial Interface
MIPI	Mobile Industry Processor Interface
CSI	Camera Serial Interface
HDMI	High-Definition Multimedia Interface
USB	Universal Serial Bus
RAM	Random Access Memory
eMMC	Embedded Multi-Media Card
microSD	Micro Secure Digital
ML	Machine Learning
TPU	Tensor Processing Unit
LPDDR	Low-Power Double Data Rate
I/O	Input/Output
LAN	Local Area Network
IoT	Internet of Things
UART	Universal Asynchronous Receiver/Transmitter
PCIe	Peripheral Component Interconnect Express
SO-DIMM	Small Outline Dual Inline Memory Module
EVE	Embedded Vision Engine
NPU	Neural Processing Unit
ONNX	Open Neural Network Exchange
SMC	Secure Multiparty Computation
Caffe	Convolutional Architecture for Fast Feature Embedding
BAIR	Berkeley AI Research
FAIR	Facebook AI Research
NGC	NVIDIA GPU Cloud
OVC	OpenVINO Model Converter
OTA	Over The Air
CI/CD	Continuous Integration/Continuous Deployment
